# Supplementary material for: Circuits for integrating learned and innate valences in the insect brain
Source: eLife. 2021 Nov 10;10:e62567. doi: 10.7554/eLife.62567 (PMC8616581; doi:10.7554/eLife.62567)
Supplement: Transparent reporting form [file elife-62567-transrepform1.pdf]

Westbrook Centre
Milton Road
Cambridge CB4 1YG, UK
P 01223 855340
W elifesciences.org
T @elife

eLife's transparent reporting form

We encourage authors to provide detailed information *within their submission* to facilitate the interpretation and replication of experiments. Authors can upload supporting documentation to indicate the use of appropriate reporting guidelines for health-related research (see EQUATOR Network), life science research (see the BioSharing Information Resource), or the ARRIVE guidelines for reporting work involving animal research. Where applicable, authors should refer to any relevant reporting standards documents in this form.

If you have any questions, please consult our Journal Policies and/or contact us: editorial@elifesciences.org.

Sample-size estimation

- You should state whether an appropriate sample size was computed when the study was being designed
- You should state the statistical method of sample size computation and any required assumptions
- If no explicit power analysis was used, you should describe how you decided what sample (replicate) size (number) to use

Please outline where this information can be found within the submission (e.g., sections or figure legends), or explain why this information doesn't apply to your submission:

For behavior datasets, *ca*. 10 repeats of experiments with groups of *ca*. 30 larvae were done, yielding a high sample size of *ca*. 300 individuals. A sample size of 10 is also used in Gershow *et al*., Nature Methods, 2012 (doi: 10.1038/nmeth.1853), where this methodology was described for the first time.

For imaging datasets, 4 to 8 repeats of each type of stimulations were done for each animal, with a sample size of 6 to 10 animals in total. Thes sample sizes are comparable to other similar type of experimental design. For example, Hige et al, Nature, 2015 (https://doi.org/10.1038/nature15396), record calcium responses of between 4 and 10 MB output neurons and compare the variability of these responses between animals possessing or lacking the learning-related gene rutabaga.

Replicates

- You should report how often each experiment was performed
- You should include a definition of biological versus technical replication
- The data obtained should be provided and sufficient information should be provided to indicate the number of independent biological and/or technical replicates
- If you encountered any outliers, you should describe how these were handled
- Criteria for exclusion/inclusion of data should be clearly stated
- High-throughput sequence data should be uploaded before submission, with a private link for reviewers provided (these are available from both GEO and ArrayExpress)

eLife Sciences Publications, Ltd is a limited liability non-profit non-stock corporation incorporated in the State of Delaware, USA, with company number 5030732, and is registered in the UK with company number FC030576 and branch number BR015634 at the address Westbrook Centre, Milton Road, Cambridge CB4 1YG.

Please outline where this information can be found within the submission (e.g., sections or figure legends), or explain why this information doesn't apply to your submission:

The number of repeats is reported in the captions for each figure and the individual datapoints are displayed in the main figure.

Reasons for excluding *in vivo* imaging datasets are given in the methods part.

eLife Sciences Publications, Ltd is a limited liability non-profit non-stock corporation incorporated in the State of Delaware, USA, with company number 5030732, and is registered in the UK with company number FC030576 and branch number BR015634 at the address Westbrook Centre, Milton Road, Cambridge CB4 1YG.

Statistical reporting

- Statistical analysis methods should be described and justified
- Raw data should be presented in figures whenever informative to do so (typically when N per group is less than 10)
- For each experiment, you should identify the statistical tests used, exact values of N, definitions of center, methods of multiple test correction, and dispersion and precision measures (e.g., mean, median, SD, SEM, confidence intervals; and, for the major substantive results, a measure of effect size (e.g., Pearson's r, Cohen's d)
- Report exact p-values wherever possible alongside the summary statistics and 95% confidence intervals. These should be reported for all key questions and not only when the p-value is less than 0.05.

Please outline where this information can be found within the submission (e.g., sections or figure legends), or explain why this information doesn't apply to your submission:

The details of the statistical test used is given in the caption of the figures.

For connectivity matrix of the neurons, Supplementary File 1 provides the raw number of synapses reconstructed between each neuron type.

For imaging data shown in Figures 4, 5 and 6, individual response to each repeat is displayed for each individual in their corresponding supplement figures. The p-value following Wilcoxon rank test, the detailed results from ANOVA, including the degree of freedom, are provided in Supplementary File 2.

Source data files are provided for each dataset shown in the manuscript regarding behavior and imaging, except for experiments where a high number of individuals (at least 100 per condition) were used to extract parameters (Figure 1b-c, 6f and 5c) and where the identity of the individual is not kept throughout the experiment.

(For large datasets, or papers with a very large number of statistical tests, you may upload a single table file with tests, Ns, etc., with reference to sections in the manuscript.)

Group allocation

- Indicate how samples were allocated into experimental groups (in the case of clinical studies, please specify allocation to treatment method); if randomization was used, please also state if restricted randomization was applied
- Indicate if masking was used during group allocation, data collection and/or data analysis

Please outline where this information can be found within the submission (e.g., sections or figure legends), or explain why this information doesn't apply to your submission:

For each figure, experimental groups were tested in a randomised way and each group was tested over multiple days to avoid possible artefact due to the day or time of the experiment.

Additional data files ("source data")

- We encourage you to upload relevant additional data files, such as numerical data that are represented as a graph in a figure, or as a summary table
- Where provided, these should be in the most useful format, and they can be uploaded as "Source data" files linked to a main figure or table

eLife Sciences Publications, Ltd is a limited liability non-profit non-stock corporation incorporated in the State of Delaware, USA, with company number 5030732, and is registered in the UK with company number FC030576 and branch number BR015634 at the address Westbrook Centre, Milton Road, Cambridge CB4 1YG.

- Include model definition files including the full list of parameters used
- Include code used for data analysis (e.g., R, MatLab)
- Avoid stating that data files are "available upon request"

Please indicate the figures or tables for which source data files have been provided:

Source data files are provided for Figure 2, 3, 4, 5, and 6.

Matlab codes for image analyses are available at
https://github.com/eschbachc/Matlab_codes_for_image_analyses

eLife Sciences Publications, Ltd is a limited liability non-profit non-stock corporation incorporated in the State of Delaware, USA, with company number 5030732, and is registered in the UK with company number FC030576 and branch number BR015634 at the address Westbrook Centre, Milton Road, Cambridge CB4 1YG.
